# Aluminosilicate Nanotubes Embedded Polyamide Thin Film Nanocomposite Forward Osmosis Membranes with Simultaneous Enhancement of Water Permeability and Selectivity

**DOI:** 10.3390/polym11050879

**Published:** 2019-05-14

**Authors:** She-Ji Shi, Ye-Han Pan, Shao-Fei Wang, Zheng-Wei Dai, Lin Gu, Qing-Yun Wu

**Affiliations:** 1 Faculty of Materials Science and Chemical Engineering, Ningbo University, Ningbo 315211, China; 1575746280@163.com (S.-J.S.); 15067131545@163.com (Y.-H.P.); wsf19941216@163.com (S.-F.W.); 2 College of Material and Textile Engineering, Jiaxing University, Jiaxing 314001, China; zwdai1981@163.com; 3 Key Laboratory of Marine Materials and Related Technologies, Ningbo Institute of Materials Technology and Engineering, Chinese Academy of Sciences; Ningbo 315201; China; gulin1985@gmail.com

**Keywords:** forward osmosis, imogolite nanotubes, polyamide, water permeability, selectivity

## Abstract

Nanocomposite membranes are strongly desired to break a trade-off between permeability and selectivity. This work reports new thin film nanocomposite (TFN) forward osmosis (FO) membranes by embedding aluminosilicate nanotubes (ANTs) into a polyamide (PA) rejection layer. The surface morphology and structure of the TFN FO membranes were carefully characterized by FTIR, XPS, FESEM and AFM. The ANTs incorporated PA rejection layers exhibited many open and broad “leaf-like” folds with “ridge-and-valley” structures, high surface roughness and relatively low cross-linking degree. Compared with thin film composite (TFC) membrane without ANTs, the TFN membrane with only 0.2 w/v% ANTs loading presented significantly improved FO water permeability, selectivity and reduced structural parameters. This promising performance can be mainly contributed to the special ANTs embedded PA rejection layer, where water molecules preferentially transport through the nanochannels of ANTs. Molecular dynamic simulation further proved that water molecules have much larger flux through the nanotubes of ANTs than sodium and chloride ions, which are attributed to the intrinsic hydrophilicity of ANTs and low external force for water transport. This work shows that these TFN FO membranes with ANTs decorated PA layer are promising in desalination applications due to their simultaneously enhanced permeability and selectivity.

## 1. Introduction

The global water crisis and fresh water shortage have become one of the most essential issues in the world [1]. It is of great demand to develop alternative water sources, such as seawater desalination and wastewater reclamation [2]. Recently, forward osmosis (FO) has drawn much attention as an emerging membrane separation technology for available desalination, because of its zero external pressure, low energy input, acceptable fouling resistance and high water recovery [3,4,5,6,7,8]. In a typical FO process, FO membranes play an important role on transporting water molecules and selectively rejecting the salt solute. Although great efforts have been devoted to improving the morphology and performance of FO membranes, it is still a challenge to simultaneously enhance their water permeability and selectivity, which are a ubiquitous trade-off. 

In nature, biological membranes are virtually able to transport water molecules but exclude solutes in cells showing high permeability and high selectivity [9]. This is attributed to specialized membrane proteins acting as intermediates across the cell membranes, possessing well-defined channel pore size combined with appropriate intermolecular forces such as electrostatic force, van der Waals, and so on [10]. However, the usage of these natural proteins inevitably suffers from their complicate membrane fabrication, high cost of production, and low stability [11]. Thus far, the development of bioinspired artificial water channel analogs has been greatly motivated as an alternative [12,13,14]. Nanoparticles with special pore structures are usually incorporated into the polyamide rejection layer to enhance the performance of the thin film nanocomposite (TFN) membranes, including metal-organic framework [15], graphene oxide [16], titanate nanotubes [17], halloysite nanotubes [18], and so on. Among others, Carbon nanotubes (CNTs) are the most popular candidate because they possess diameters in the nanometer range (1–2 nm), unique tubular geometry, and atomically smooth surfaces that allow the ultra-fast transport of water molecules [19,20,21,22,23]. Thus, CNTs have been widely applied to composite membranes in desalination applications [22,23,24,25,26,27,28]. Song et al. [24] incorporated CNTs into the rejection layers of FO membranes, and the obtained thin film nanocomposite (TFN) FO membranes presented 54% enhancement in water flux. Moreover, the embedded CNTs even improved the solute rejection and selectivity when the CNTs content increased to 0.05 wt %. Similarly, Li et al. [29] incorporated the sulfonated CNTs into the PA layer, resulting in the enhancement of permeability and selectivity of TFN FO membranes. However, CNTs as hydrophobic nanotubes are restrictive due to three main reasons: 1) high synthesis cost for high-purity monodispersed structure, 2) complicated surface modification on the external surface of CNTs, 3) difficult interior functionalization through covalent bonds. Therefore, it would be attractive to develop intrinsically hydrophilic artificial water channel analogs. 

Aluminosilicate nanotubes (ANTs), known as one dimensional single-walled hydrophilic nanotubes, have gained particular attention in recent years [30,31,32,33]. They are synthesized from inexpensive metal oxide under hydrothermal or solvothermal conditions with the general formula of (OH)_3_Al_2_O_3_SiOH [34]. The resulted ANTs have high monodispersity in terms of pore diameters of 1–2 nm, which are comparable to that of CNTs. However, unlike the inert CNTs, abundant hydroxyl groups are bound on the inner and outer walls of ANTs, endowing the nanotubes with good hydrophilicity and thus good dispersion in aqueous solutions. Furthermore, their composition and functionality are tunable by using different precursors, even for the interior surface [35]. These unique features make ANTs one of the most promising competitors of CNTs for the potential applications in desalination [31,36,37]. Kang et al. [38] incorporated ANTs into poly(vinyl alcohol) matrix, and the resulted nanocomposite membranes enhanced the water permeability as increasing ANTs volume fraction. Barona et al. [36] added 0.59 wt % ANTs into the polyamide layer of reverse osmosis (RO) membranes, leading to the increase in both water permeability and salt rejection. This result was explained in terms of the electrostatic repulsion and steric hindrance of ANTs. Recently, Li et al. [39] also promoted the water permeability of surface modified imogolite nanotubes incorporated TFN RO membranes for 2.4 times higher than TFC membranes. The enhanced water permeability by ANTs has also been attracting attentions and motivating the studies of water diffusion in ANTs from the aspect of molecular scale. Scalfi et al. [40] described a strong structuration of the water confined in the nanotube, with specific adsorption sites and a distribution of hydrogen bond patterns. Zang et al. [32] studied the transport properties of water, methanol, and ethanol through a single-walled aluminosilicate nanotube and showed that water diffusion in ANTs is comparable to bulk phase due to the large pore diameters. These works described a clear physical image for the structure and self-diffusion behavior of water molecules in ANTs. Nonetheless, several questions were still not well addressed: (1) What are the effects of ANTs on the water permeability and selectivity of TFN membranes in FO process without any hydraulic pressure? (2) What are the competitive diffusion behaviors of water molecules and the ions (such as sodium cation and chloride anion) in ANTs? The answers to these questions offer key information to develop novel TFN FO membranes, and to comprehensively understand the mechanism of FO process using ANTs as water channels. 

In this paper, novel TFN membranes were fabricated by incorporating the hydrophilic ANTs into the PA rejection layer during the interfacial polymerization. Typically, the effects of ANTs on the morphology and performance of TFN membranes were investigated by embedding different contents of the synthesized ANTs. It should be pointed out that the ANTs embedded PA rejection layer endowed the TFN membranes with both enhanced water permeability and solute rejection due to the characteristic physicochemical structures of ANTs. Furthermore, molecular dynamics simulation was applied to study the competitive transport of water molecules, sodium cations, and chloride anions through the nanotubes. These ANTs embedded TFN FO membranes could be promising in desalination application.

## 2. Experimental

### 2.1. Materials

Polysulfone (PSf, *M*_n_ = 22,000) as the substrate material was purchased from Solvey Co., Shanghai, China. *N*, *N*-Dimethylacetamide (DMAc, >99%) and polyethylene glycol (PEG, *M*_n_ = 400) were supplied by Sinopharm Chemical Reagent Co. Ltd., Shanghai, China, and used as the solvent and additive for the substrate preparation, respectively. Aluminum nitrate nonahydrate (Al(NO_3_)_3_⋅9H_2_O, AR, Aladdin) and tetraethyl orthosilicate (TEOS, Sinopharm Chemical Reagent Co. Ltd., Shanghai, China) were chosen to synthesize the aluminosilicate nanotubes (ANTs). *m*-Phenylenediamine (MPD, >99%, Acros Organics, Shanghai, China) and trimesoyl chloride (TMC, ~98%, Aladdin, Shanghai, China) were used for the interfacial polymerization (IP) to form the polyamide (PA) rejection layer. Sodium hydroxide (NaOH), ammonium hydroxide (NH_3_⋅H_2_O), sodium hypochlorite (NaClO), sodium bisulphite (NaHSO_3_) and sodium chloride (NaCl) were all supplied by Sinopharm Chemical Reagent Co. Ltd., Shanghai, China.

### 2.2. Synthesis and Characterization of ANTs

The synthesis of ANTs was according to the method reported by Arancibia-Miranda et al [41]. Al(NO_3_)_3_⋅9H_2_O, TEOS and NaOH were used as main sources of ANTs. Acertain amount of TEOS was mixed with an aqueous solution of 5 mM Al(NO_3_)_3_⋅9H_2_O until the final Si/Al mole ratio was 0.5. After stirred vigorously for 1 h, 1×10^−2^ M NaOH solution was dripped into the solution to obtain an Al:Si:OH mole ratio of 2:1:4. The resulting clear solution reacted at 95 °C for 7 days. When the solution was cooled down to room temperature, its pH was adjusted to 8 with 0.1 M NH_3_⋅H_2_O to yield a transparent gel. The gel was washed with deionized water and separated by centrifuging at 9000 rpm for 15 min, and then dried in a vacuum oven at 60 °C overnight. The dried samples were milled into powders.

Fourier transform infrared (FTIR, Nicolet 6700, Thermo Fisher Scientific, Shanghai, China) spectroscopy was conducted to confirm the nanotube synthesis. The spectra were collected in the range of 400~4000 cm^−1^ by cumulating 64 scans with a resolution of 2 cm^−1^. X-ray diffraction (XRD, D8 Advance Davinci, Bruker, Karlsruhe, Germany) analysis was applied to confirm the crystalline of ANTs with Cu: Kα source (*λ* = 1.54 Å) over the 2*θ* range of 3–15°. The structure of ANTs was observed by transmission electron microscope (TEM, JEOL2100, JEOL, Tokyo, Japan) and field emission scanning electron microscope (FESEM, SU4800, Hitachi, Tokyo, Japan). The pore size distribution was analyzed by nitrogen adsorption technique with automated gas sorption analyzer (ASAP2020HD88, Micromeritics, Norcross, GA, USA). This experiment was performed at 77 K, and the powder was first degassed for 10 h at 353 K. The total pore volumes corresponding to the micropore volumes were calculated by the amounts of N_2_ adsorbed at relative pressures. Horvath–Kawazoe method was used to calculate the pore size distribution of the nanotubes [42].

### 2.3. Fabrication and Characterization of Forward Osmosis (FO) Membranes

#### 2.3.1. Preparation of PSf Substrates

The dope solution was prepared by dissolving 15 wt% PSf and 5 wt% PEG in DMAc, and then stirred at 60 °C for 8 h. After degassing, the dope solution was cast on a clean glass plate, and subsequently immersed into a water coagulation bath containing 0.3% (v/v) DMAc to initiate a phase inversion process at room temperature. The obtained PSf membranes were transferred to another water bath and stored for at least 1 day to remove the residual solvent before used as the substrates of TFN FO membranes. 

#### 2.3.2. Preparation of Thin Film Nanocomposite (TFN) Membranes

PA rejection layers were prepared by interfacial polymerization (IP) process on the top of the PSf substrates. The PSf substrate was fixed in a special container with the effective area of 12.56 cm^2^, and then the 3.4 wt % MPD solution was poured onto the substrate surface. After maintaining for 2 min, the residual MPD on the substrate was removed. Then, the 0.15 wt % TMC solution in isopar-G with different contents of ANTs (0, 0.08, 0.2 and 0.5 w/v%) was poured onto the amine penetrated substrate for 1 min. The organic solution containing ANTs was sonicated for10 min before pouring onto the substrate to relieve nanotube agglomeration. The PA layer was formed after cured in a vacuum oven at 90 °C for 8 min. After that, the composite membrane was successively rinsed with a 200 ppm NaClO aqueous solution for 2 min and a 1000 ppm NaHSO_3_ aqueous solution for 30 s. The membrane was again heated in a vacuum oven at 90 °C for 2 min, because the pre- or post-treatment of the membranes have great effects on their morphology and performance [43,44,45]. At last, the obtained FO membranes were stored in DI water at room temperature. According to the quantity of the used ANTs, these synthesized FO membranes were denoted as TFC, TFN 0.08, TFN 0.2, and TFN 0.5.

#### 2.3.3. Membrane Characterization

ATR-FTIR spectroscope (Nicolet 6700, Thermo Fisher Scientific, Thermo Fisher Scientific, Shanghai, China) was used to determine the functional groups in TFC or TFN membranes. The surface and cross-section morphologies of membranes were observed by FESEM. X-ray photoelectron spectroscopy (XPS, Kratos AXIS ULTRA, Kratos, Shanghai, China) was used to examine the elemental composition on the surface of TFC or TFN membrane. Surface roughness of membranes with different ANTs loading was quantitatively measured by atomic force microscope (AFM, Dimension 3100, Veeco, Shanghai, China), which used silicon AFM probes with the spring constant of 40 N/m.

### 2.4. Evaluation of Membrane Performance

Water permeability coefficient (*A*), salt rejection (*R*_s_) and salt permeability coefficient (*B*) of the prepared TFC or TFN membranes were evaluated using a cross flow reverse osmosis (RO) filtration setup. The effective membrane area (*S*_m_) was 4.91 cm^2^. In this study, an aqueous solution of 20 mM NaCl was used as a feed solution under an operating pressure (*ΔP*) of 5 bar with velocity of 3.0 L/min. The following equations were applied to calculate these intrinsic transport properties:

(1) $$J_{v}=\frac{\Delta V}{\Delta tS_{m}}$$

(2) $$A=\frac{J_{v}}{\Delta P}$$

(3) $$R_{S}=\frac{C_{f}-C_{p}}{C_{f}}\times 100\%$$

(4) $$B=A\times \frac{\left(1-R_{S}\right)\cdot \left(\Delta P-\Delta \pi \right)}{R_{S}}$$

where Δ*V* and Δ*t* represent the volume of permeation water and the measuring time, respectively. *C*_f_ and *C*_p_ refer to the salt concentrations in the feed and permeate solution, respectively and they were all determined by conductivity measurement. Δ*π* is the osmotic pressure difference across the membrane.

The laboratory-scale cross-flow cell of FO setup had an effective membrane area of 4.9 cm^2^. The feed and draw solutions were DI water and 1 M NaCl solution, respectively. Above-mentioned solutions flowed across the chambers with similar flow rates of 40 L/h and their temperature was maintained at 25 ± 1 °C. FO tests were performed in both membrane orientations, i.e., active layer facing feed water (FO mode, AL-FS)and active layer facing draw solution (PRO mode, AL-DS) orientations. The water permeation flux (*J*_w_) was determined by the equation (5):

(5) $$J_{w}=\frac{\Delta m/\rho }{\Delta tS_{m}}$$

where Δ*m* is the weight change of feed solution, *S*_m_ is the effective area of membrane, *ρ* is the density of feed solution and Δ*t* is the measuring time.

The reverse solute flux (*J*_s_) of FO membrane was calculated from the change of salt content in feed solution based on conductivity measurement. It was calculated by the following equation:

(6) $$J_{S}=\frac{\Delta \left(C_{t}V_{t}\right)}{\Delta tS_{m}}$$

where *C*_t_ and *V*_t_ are the salt concentration and the volume of feed solution based on the time interval, respectively.

The structural parameter (*S*) can be calculated as follows:

(7) $$S=\frac{D}{J_{w}}\ln\left(\frac{B+A\times \pi_{D}}{B+J_{w}+A\times \pi_{F}}\right)$$

where *π*_D_ and *π*_F_ are the osmotic pressures of the draw solution and feed solution, respectively, and *D* is the solute diffusion coefficient in water.

### 2.5. Molecular Dynamics Simulation

Molecular dynamic simulation was carried out on GROMACS [46] using CHARMM [47] force field. The force field parameters and initial structure of ANTs were achieved from literature [48]. The ANTs were placed between two carbon walls, forming a membrane-like structure. The three-point water model of TIP3P was used in the simulation [49]. First, water molecules were placed at one side of the membrane, and then randomly replaced by sodium cations and chloride anions, to get a salt solution of 1 M. The system ran in NVT ensemble at 300K for 20 ns to investigate the permeation of salt solution into ANTs. Second, water molecules were filled into the ANTs, as well as placed at both sides of the membrane, and then equilibrated under NVT ensemble at 300 K for 10 ns. Third, object molecules (the water molecule, sodium cation, and chloride anion) were placed at one side of the membrane and pushed into the ANTs by an external force with the umbrella sampling method, with the pulling speed of 0.01 nm/ns, and pulling constant of 1000 kJ/(mol⋅nm^2^).

## 3. Results and Discussion

### 3.1. Structure and Chemical Composition of Synthesized ANTs

**Figure 1:**
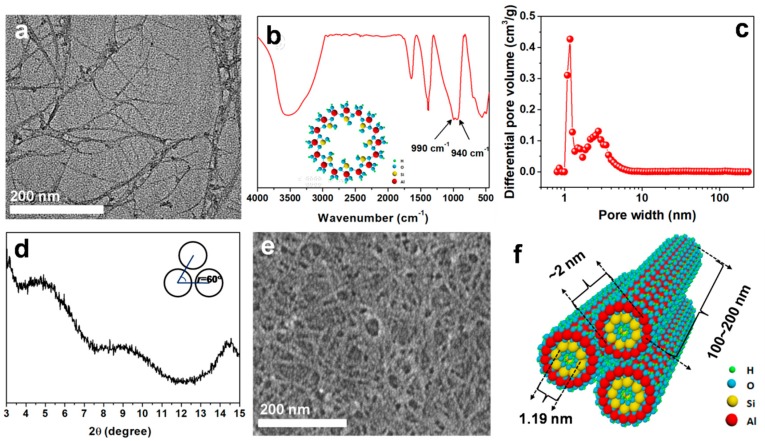
Characterization of synthesized aluminosilicate nanotubes (ANTs): (**a**) TEM image, (**b**) FTIR spectrum, (**c**) BET curve, (**d**) XRD pattern, (**e**) FESEM image, and (**f**) schematic illustration of ANTs. Insert in (b) is the atom structure of ANTs viewed along the axial direction.

The typical structure of ANTs has been first confirmed by TEM, as shown in Figure 1a. It is clear that the synthesized aluminosilicate are presented in the form of thin fibers. Their lengths are approximately 100–200 nm and the average outer diameters are roughly 2 nm, which are in accordance with Farmer et al. [34]. FTIR spectrum was used to characterize the chemical composition of the synthetic ANTs (Figure 1b). The wide band around 3500 cm^−1^ is attributed to the large amount of OH groups on nanotubes, implying their intrinsic hydrophilicity. Bands at 570 cm^−1^ and 693 cm^−1^ are associated with the stretching vibration of the O-Si-O and O-Al-O groups, respectively. Remarkably, the peaks at 990 cm^−1^ and 940 cm^−1^ corresponding to the Si-O stretching vibrations become the typical features of the tubular structure of the ANTs [50,51,52].

This hollowed structure can be proved by BET curve (Figure 1c). It demonstrates that most of the synthesized ANTs possess effective pore diameter is 1.19 nm, which is in good agreement with those reported by Barona et al. [36]. Moreover, the XRD pattern also reveals the information of the tubular structures from the diffraction peaks at 2*θ* < 20° [53]. Figure 1d shows two characteristic diffraction peaks at 2*θ* of 4.7° and 8.7°, corresponding to the *d*-values of 1.88 and 1.01 nm, respectively. It indicates that the inner diameter of ANTs is around 1 nm, and the outer one is about 2 nm, which are in consistent with Farmer et al. [34]. 

On the other hand, another peak exists around 2.58 nm in BET curve, which may be ascribed to the pores from the stacked ANTs. Although it is accepted that ANTs have good dispersion in water, ANTs may stack into bundling when directly dried from the suspension system with high concentration. The bundling arrangement also can be proved by the shoulder diffraction peaks at 2*θ* of 5.9° in XRD pattern (Figure 1d), which suggests that the ANTs in the powders form into triangular bundles [54]. Furthermore, Figure 1e shows the morphology of ANTs after drying, which is much thicker than those dispersed in solvents.

### 3.2. Surface Composition and Morphology of TFN Membranes

**Figure 2:**
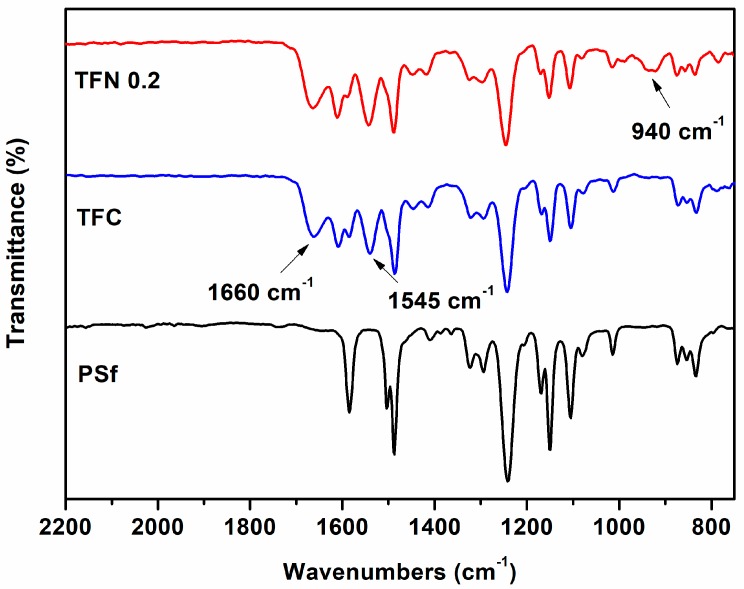
ATR-FTIR spectra of PSf substrate, thin film composite (TFC) membrane and thin film nanocomposite (TFN) 0.2 membrane.

ATR-FTIR spectra were carried out to investigate the surface chemical composition of membranes. As shown in Figure 2, all the membranes have the similar peaks at 1151 cm^−1^ (symmetric O=S=O stretching), 1244 cm^−1^ (asymmetric C-O-C stretching), 1297 cm^−1^ (asymmetric O=S=O stretching), 1491 cm^−1^ (C=C aromatic ring stretching) and 1588 cm^−1^ (CH_3_-C-CH_3_ stretching), which attribute to the specific functional groups of PSf substrates [55]. In contrast, two significant peaks for TFC and TFN 1.0 membranes at 1660 cm^−1^ and 1545 cm^−1^ represent the C=O stretching (amide Ι band) and C-N stretching (amide Ⅱ band), respectively [56]. These two peaks are characteristic of PA, and reveal the occurrence of interfacial polymerization to TFC and TFN membranes. In contrast, an extra peak at 940 cm^−1^ (Si-O stretching) appears in the spectrum of TFN membrane, which belongs to the characteristics of ANTs [50,51,52]. This peak is a strong evidence for successfully incorporating ANTs into PA layer.

The chemical composition of the membrane surface was further determined by XPS, and the results are summarized in Table 1. The existence of ANTs in the PA layer can be verified by the unique Al element in ANTs. As can be seen, the content of Al element in TFN membrane surface increases with the incorporation of ANTs, while there is no Al can be detected in TFC membrane. This result suggests the successful incorporation of ANTs in the surface of TFN membrane. Generally, the atomic ratio of O/N is commonly used to investigate the cross-linking degree of PA thin film [57]. Due to the extra oxygen amount brought in by ANTs, C/N ratio is considered to evaluate the cross-linking degree of PA rejection layer [58]. According to the finding of Lind et al. [57], a fully cross-linked PA has a theoretical C/N ratio of 6. Compared to TFC membrane, TFN membranes present relatively high C/N ratios indicating their low cross-linking degree. It could be concluded that the incorporation of ANTs may alter the PA structure and then further affect the performance of TFN membranes.

**Figure 3:**
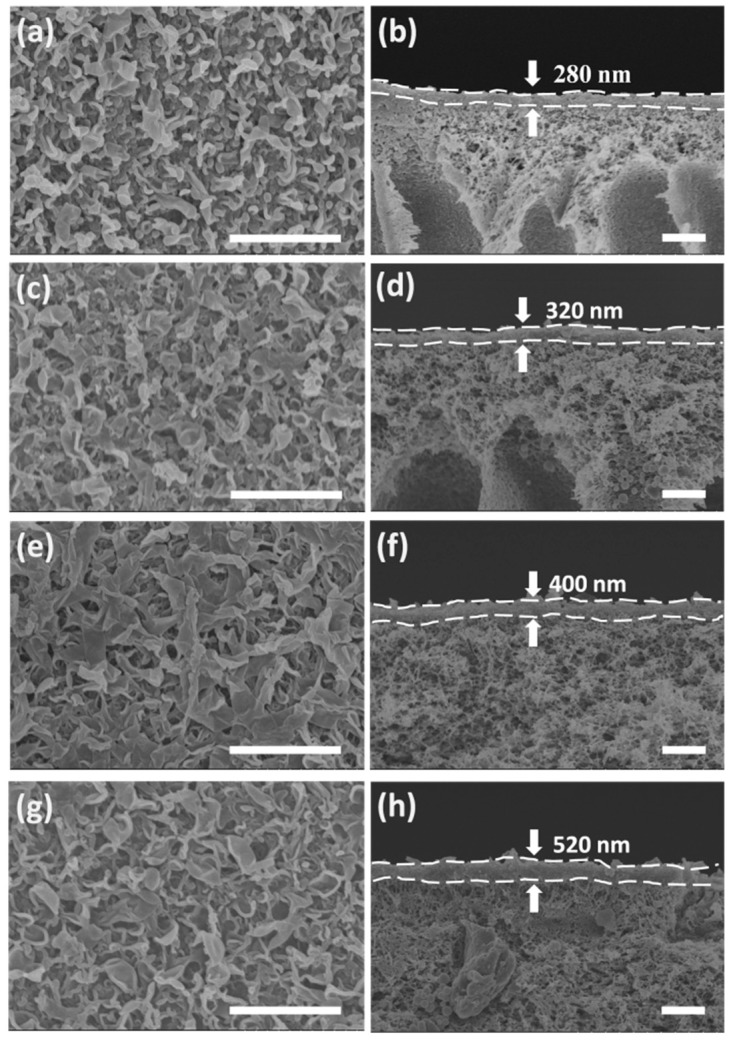
FESEM micrographs showing the surfaces (Left) and cross-sections (Right) of (**a,b**) TFC, (**c,d**) TFN 0.08, (**e,f**) TFN 0.2 and (**g,h**) TFN 0.5. (Scale bars are 2 μm).

The surface and cross-section morphology of the TFC and TFN membranes are shown in Figure 3. The top surface of TFC membrane exhibits typical “ridge-valley” morphology, whereas TFN membranes have more open and broader “leaf-like” structures with the incorporation of nanotubes. This surface structure enlarges the contact area between feed solution and TFN membranes, and thus enhances the water permeability. In addition, the thickness of the PA rejection layer was measured according to the cross-section morphology of TFC and TFN membranes. The results show that the thickness of the PA layer increases with the ANTs loading. It is well known that MPD diffuses into organic phase and reacts with TMC during the IP process [59]. This process is very fast and the initial formed PA film will block the further diffusion of MPD [60]. When the ANTs is added into the organic phase, the hydrophilic nanotubes will increase the miscibility of the aqueous and organic phase, and expands the reaction zone [17].

**Figure 4:**
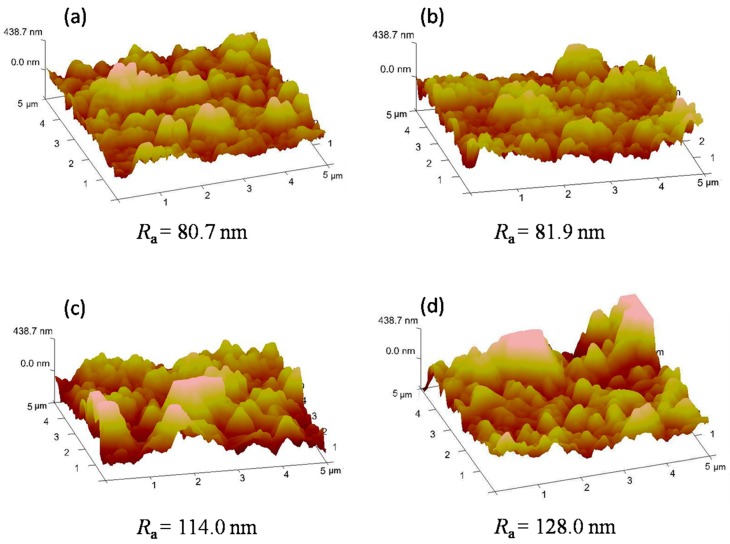
AFM images of polyamide (PA) rejection layers with different ANTs loading of (**a**) TFC, (**b**) TFN 0.08, (**c**) TFN 0.2 and (**d**) TFN 0.5 membranes.

Figure 4 shows the 5 µm × 5 µm three-dimensional AFM images of the membranes. Typical “ridge-and-valley” structures are observed on the surfaces of all membranes. It is worth noting that compared to TFC membrane, TFN membranes with ANTs present obvious “leaf-like” folds, which correspond to the structures seen in FESEM images (Figure 3). According to the *R*_a_ values, it is obvious that incorporating ANTs into a PA layer may increase the surface roughness of TFN membranes.

### 3.3. Effect of ANTs Loading on Intrinsic Transport Performance of TFN Membranes

Table 2 lists the water permeability coefficient (*A*), the salt permeability coefficient (*B*) and the salt rejection (*R*_s_) of TFC and TFN membranes, which are generally used to represent the intrinsic transport performance of FO membranes. The control TFC membrane shows the *A* value of 0.26 L/m^2^ h bar, while TFN 0.08, TFN 0.2 and TFN 0.5 exhibit 0.59, 0.66 and 2.15 L/m^2^ h bar, which are 227%, 254% and 827% higher than that of the control membrane, respectively. This means the incorporation of ANTs into the PA layer effectively improves the water permeability of TFN membranes. The improvement of water permeability for TFN membranes could be attributed to two reasons. On the one hand, the existence of ANTs in the PA layer may bring two kinds of nanochannels including the inner cores of the nanotubes and the interfacial gap between ANTs and the polymer matrix, which could create more chance for water molecules to pass through the membrane. On the other hand, hydrophilic ANTs may increase the miscibility of the aqueous and organic phases by releasing heat during the IP process [58]. The resulted broad “leaf-like” structure increases the contact areas between the composite membrane and the feed solution, resulting in the improved water permeability. 

Moreover, the salt rejection can also be enhanced when ANTs loading increases. *R*_s_ value increases from 61.17% of TFC to 86.67% of TFN0.2 membrane. It suggests that the ANTs incorporated PA layer owns good water permeability without sacrificing salt rejection. It is interesting since it was usually a trade-off between water permeability and salt rejection in the reported cases of nanocomposite membranes [18]. It is also true for the CNTs-embedded PA layers [24]. As ANTs loading further increases to 0.5 wt %, the TFN 0.5 presents a relatively low *R*_s_ value (48.43%). It may be explained by the defects formed by ANTs aggregation at high concentration during the IP process. The selectivity of membranes during the FO process can be directly reflected by the term of salt permeability/water permeability (*B/A*) ratio. The small *B/A* ratio implies weak solute reverse diffusion from the draw solution into the feed solution. From the data, it is worthwhile to note that TFN 0.2 membrane has a low *B/A* ratio and high water permeability, which can be regarded as the optimal membrane for FO applications.

### 3.4. Effect of ANTs Loading on FO Performance of TFN Membranes

**Figure 5:**
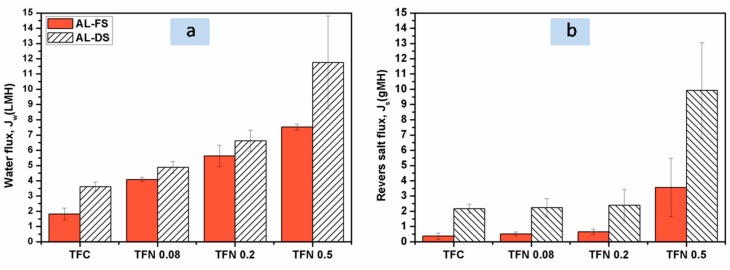
Forward osmosis (FO) performance tests: (**a**) water fluxes, and (**b**) reverse salt fluxes across the FO membranes. Experimental conditions: 25 °C, 1 M NaCl as the draw solution, DI water as the feed solution, and cross-flow velocities of 40 L/h on both sides of the membranes. Data were obtained from at least three tests on independent samples.

The FO performance of the composite membranes was measured in both “AL-FS” and “AL-DS” modes using 1M NaCl aqueous solution as the draw solution and the DI water as the feed solution in a conventional FO flow cell. Figure 5a illustrates that all the composite membranes show higher water fluxes in AL-DS orientation than those in AL-FS orientation. The range of water fluxes changes from 1.82 ± 0.39 ~7.52 ± 0.2 L/m^2^h in AL-FS to 3.62 ± 0.29 ~11.76± 3.04 L/m^2^h in AL-DS because the ICP effect in AL-DS mode is concentrative which is considered to be weaker than the alternative situation [61,62]. It also can be seen that all TFN membranes containing different ANTs loading exhibit higher water fluxes than the control TFC membrane in both orientations. For example, the water flux of TFN 0.2 (5.63 ± 0.7 L/m^2^h) is three times as large as that of TFC (control) (1.82 ± 0.39 L/m^2^h) in AL-FS mode. When adding 0.5 w/v% ANTs, the water flux of TFN 0.5 further increases to 7.52 ± 0.2 L/m^2^h in AL-FS mode. Correspondingly, the *S* values remarkably reduce from 5.09 mm for TFC membrane to 1.36 mm for TFN 0.5 membrane (Table 2). These results suggest that the ICP effect can be alleviated by enhancing the water flux of FO membranes, which are consistent with those reported by Tang et al. [63]. More importantly, the reverse salt flux of TFN membranes remains in a low level, especially for the cases of TFN 0.08 and TFN 0.2. As shown in Figure 5b, the reverse salt fluxes of TFN 0.2 are as low as 0.66 ± 0.18 g/m^2^h in AL-FS mode and 2.4 ± 1.02 g/m^2^h in AL-DS mode, which are almost the same as those of TFC membrane. In addition, further increasing ANTs loading to 0.5 w/v% might cause some nanotubes to be severely agglomerated, and thus result in a high reverse salt flux. 

**Figure 6:**
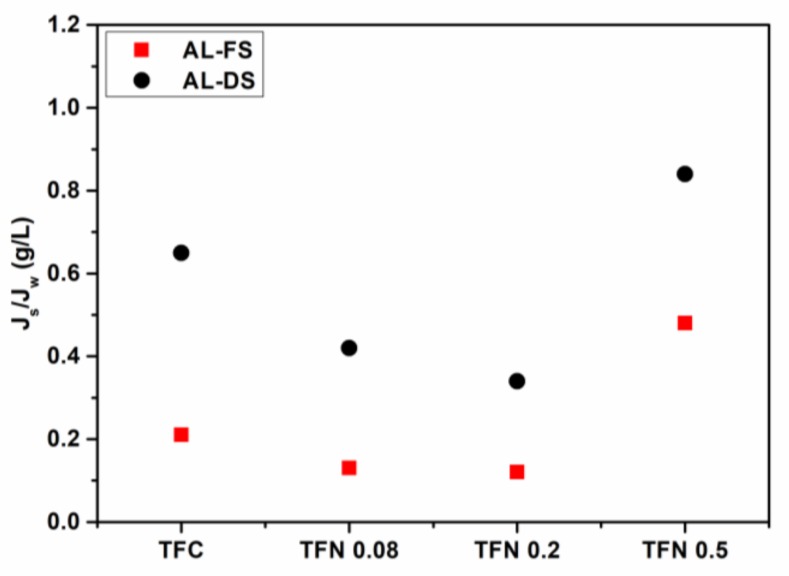
*J_s_/J_w_* values of FO membranes in AL-FS and AL-DS orientations.

In accordance with practice, the selectivity of the FO membrane was also evaluated by the ratio of the reverse salt flux to the water flux, *J_s_/J_w_*, in both AL-DS and AL-FS modes (Figure 6). Generally, a membrane with a low *J_s_/J_w_* value could exert satisfactory selectivity in rejecting solute relative to water [64]. In this study, TFN 0.2 has the lowest *J_s_/J_w_* values (0.12 g/L in AL-FS mode, and 0.34 g/L in AL-DS mode) and it is superior to some other FO membranes [65,66,67,68]. This result is inconsistent with the salt rejection as mentioned above, in which TFN 0.2 shows the highest value of 86.67%. It should be further pointed out that as the ANTs loading increases to 0.2 w/v%, the *J_s_/J_w_* value declines and meanwhile the water flux remarkably increases. It suggests that both the salt rejection and water permeability of TFN membranes can be improved by embedding appropriate contents of ANTs into the PA rejection layer, which are always a trade-off in nanocomposite membranes. This result is in accordance to that of CNTs incorporated TFN membranes reported by Song et al. [24], which may be attributed to the thin tubular structures of CNTs and ANTs (inner size is ~1 nm). In comparison, thick nanotubes, such as HNTs (the inner size is ~300 nm), facilitate to improve the water permeability of TFN membranes, but the salt rejection is always sacrificed [18]. 

### 3.5. Molecular Dynamic Simulation of Water Diffusion through ANTs

The above experimental results have proved that the introduction of ANTs into the composite membrane impressively improves its performance in the FO process. Not only the water permeability has been enhanced, but also the salt rejection is increased. The results need to be explained from the molecular scale, considering the trade-off relationship between water flux and selectivity. Herein, molecular dynamic (MD) simulations have been carried out to understand the transporting behaviors of water molecules, sodium cations as well as chloride anions in ANTs.

**Figure 7:**
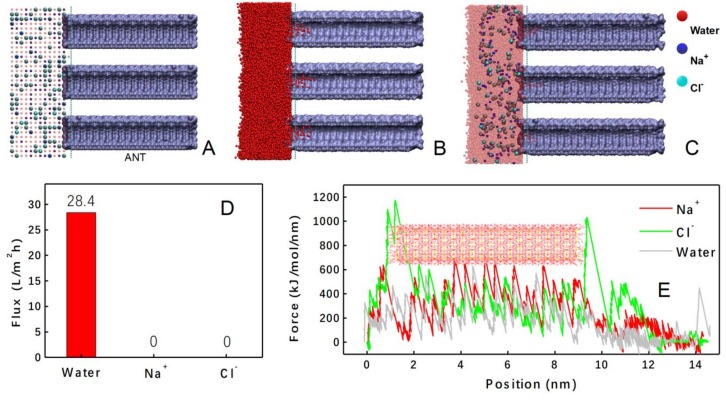
(**A**) The initial state of molecules transport into ANTs. (**B**) The state of water molecules in ANTs at 20 ns. (**C**) The state of sodium cations and chloride anions in ANTs at 20 ns. (**D**) Calculated flux of water molecules, sodium cations, and chloride anions. (**E**) The external force for water molecules, sodium cations, and chloride anions varied with the position in nanotubes.

First, water molecules with sodium and chloride ions in the density of 1 M were introduced in one side of the model membrane (Figure 7A), and the system was then run at 300 K. After 20 ns, water molecules diffused into the nanotubes along the inner wall (Figure 7B). This is because the inner walls of ANTs are attached with hydroxyl groups, which attract water molecules to get rid of the attraction from bulk phase and diffuse into the nanotubes [32]. It should be noted that the permeation of water molecules into ANTs is spontaneous, without any external driving force. The water flux through the aluminosilicate nanotubes can be calculated to be 28.4 L/m^2^h (Figure 7D).The spontaneous permeation behavior of water is a key factor to explain the increase of water flux of the TFN membrane under FO process.

On the other hand, it is interesting to find that no ions moved into the ANTs without any external driving force (Figure 7C). This is consistent with our experimental results, where high selectivity for water was found. Still, such a high selectivity of ANTs in microscopic scale for water was unexpected to us. This result may be ascribed to the different coulombic forces rather than the size exclusion effect, considering that the small radius of sodium cation (0.99Å) and chloride anion (1.81 Å), respectively [69]. Therefore, the coulombic forces have been studied by a steered molecular dynamics (SMD) simulation from an equilibrium configuration, where water molecules are fully filled into the aluminosilicate nanotube. The object molecule (water, sodium cation or chloride anion) was placed at one end of the nanotube, and then was pushed into the ANTs by an external force. The external forces for the three molecules were recorded and plotted in Figure 7E. The different external force curves should be attributed to the different interactions between the ANTs and the object molecules. The chloride anion was mostly repelled by the ANTs, and the movement of sodium cation was also more obviously prevented than that of the water molecule. As we know, abundant hydroxyl groups in the inner wall of the ANTs are negatively charged as a whole, due to the charge deviation of Si-O covalent bonds. Therefore, we can attribute the repulsive force on the chloride anion to the coulombic interaction. On the contrary, the inner wall of ANTs is attractive to the sodium cation. However, the attractive force between the sodium cation and the inner wall of ANTs is too intensive comparing with that of water. The intensive interaction will fix the sodium cation onto the inner wall once it enters the tube and stops it from further diffusion into the tube structure. 

**Figure 8:**
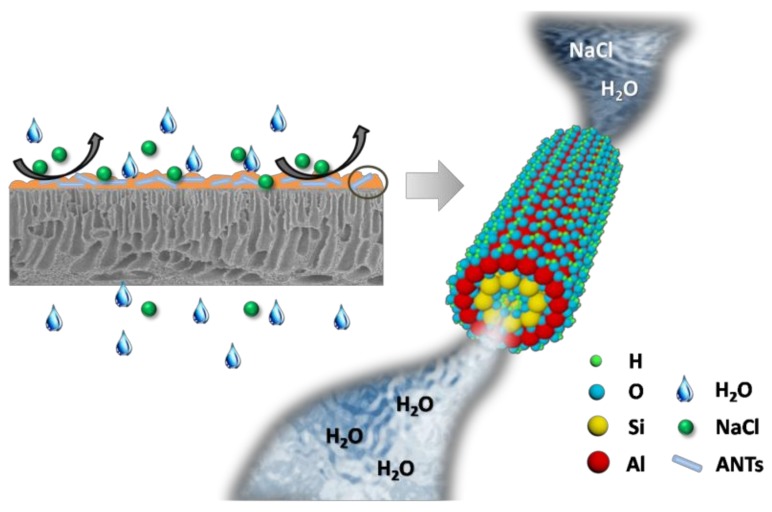
Schematic illustration for TFN membranes with ANTs embedded PA rejection layer and water transport through the inner pores of ANTs.

It can be concluded that the improved water flux and salt rejection in the TFN membrane could be attributed to the existence of ANTs in the PA layer. As shown in Figure 8, TFN membranes are composite of an ANTs embedded PA rejection layer and a substrate. The ANTs embedded PA rejection layer is responsible for the transport of water molecules and the rejection of solute: (1) The broad “leaf-like” morphology of TFN membranes have extended the contact area between water molecules and membranes; (2) ANTs may affect the interfacial polymerization process because ANTs in the organic phase may hydrate and release heat when contacted with aqueous solution [58]; (3) ANTs with appropriate contents will provide some specific nanochannels through the inner cores of the nanotubes that could be helpful to transfer water molecules and reject the salt molecules, such as 0.2 w/v% in this study. It should be avoided to adding extra ANTs which may cause severe agglomeration to form microvoids between nanotubes and the polymer matrix, and then result in high reverse salt flux. 

## 4. Conclusions

Aluminosilicate nanotubes (ANTs) as water channels were synthesized and embedded into polyamide (PA) rejection layer to form novel thin film nanocomposite (TFN) FO membranes. The incorporation of ANTs altered the structures of the PA rejection layers, and further affected the FO performance of TFN membranes. The ANTs embedded PA layers presented low cross-linking degree, broad “ridge-valley” morphology and high roughness. The incorporated ANTs remarkably promote the water permeability of TFN membranes, which effectively reduce the internal concentration polarization effect. Meanwhile, the salt rejection and selectivity of TFN membranes can be obviously enhanced when ANTs loading increases to 0.2 w/v%. The molecular dynamic simulation proved that the simultaneous improvement of water flux and salt rejection could be attributed to the selectivity of ANTs being superior to water molecules through the nanochannels. These TFN membranes are promising in effective desalination and power generation.

## Figures and Tables

**Table 1:** XPS results for thin film composite (TFC) and thin film nanocomposite (TFN) membranes.

Membrane	C (%)	O (%)	N (%)	Al (%)	Si (%)	C/N
TFC	73.19	12.51	11.49	0	2.82	6.37
TFN 0.08	71.23	17.13	9.27	0.52	1.85	7.68
TFN 0.2	71.89	14.43	10.28	0.99	2.4	6.99
TFN 0.5	72.6	14.31	9.79	1.11	2.19	7.28

**Table 2:** The intrinsic transport properties of TFC and TFN membranes.

FO membranes	*A*^a^ (L/m^2^ h bar)	*B*^b^ (L/m^2^ h)	*R_s_* (%)	*B/A* (bar)	*S* (mm)
TFC	0.26 ± 0.03	0.58 ± 0.05	67.17 ± 8.28	2.22	5.09
TFN 0.08	0.59 ± 0.20	0.79 ± 0.12	75.51 ± 2.34	1.34	2.37
TFN 0.2	0.66 ± 0.17	0.44 ± 0.08	86.67 ± 6.13	0.67	1.61
TFN 0.5	2.15 ± 0.53	9.60 ± 0.37	48.43 ± 11.66	4.47	1.36

^a^ Water permeability was measured in RO testing mode at 5 bar and DI water as feed solution. ^b^ Salt permeability was measured in RO testing mode at 5 bar and 20 mM NaCl aqueous solution as feed solution.
